# The interplay of sensory feedback, arousal, and action tremor amplitude in essential tremor

**DOI:** 10.1038/s41598-024-54528-5

**Published:** 2024-02-21

**Authors:** Julius Welzel, Miriam Güthe, Julian Keil, Gesine Hermann, Robin Wolke, Walter Maetzler, Jos S. Becktepe

**Affiliations:** 1 University Hospital Schleswig-Holstein, Kiel, Germany; 2 Department of Psychology, University of Kiel, Kiel, Germany

**Keywords:** Neurology, Diseases of the nervous system

## Abstract

Essential tremor (ET) amplitude is modulated by visual feedback during target driven movements and in a grip force task. It has not been examined yet whether visual feedback exclusively modulates target force tremor amplitude or if other afferent inputs like auditory sensation has a modulatory effect on tremor amplitude as well. Also, it is unknown whether the enhanced sensory feedback causes an increase of arousal in persons with ET (p-ET). We hypothesized that (1) amplitude of tremor is modulated by variation of auditory feedback in the absence of visual feedback in a force tremor paradigm; (2) increase of tremor amplitude coincides with pupillary size as a measure of arousal. 14 p-ET and 14 matched healthy controls (HC) conducted a computer-based experiment in which they were asked to match a target force on a force sensor using their thumb and index finger. The force-induced movement was fed back to the participant visually, auditory or by a combination of both. Results showed a comparable deviation from the target force (RMSE) during the experiment during all three sensory feedback modalities. The ANOVA revealed an effect of the high vs. low feedback condition on the tremor severity (Power 4–12 Hz) for the visual- and also for the auditory feedback condition in p-ET. Pupillometry showed a significantly increased pupil diameter during the auditory involved high feedback conditions compared to the low feedback conditions in p-ET. Our findings suggest that action tremor in ET is firstly modulated not only by visual feedback but also by auditory feedback in a comparable manner. Therefore, tremor modulation seems to be modality independent. Secondly, high feedback was associated with a significant pupil dilation, possibly mirroring an increased arousal/perceived effort.

## Introduction

Tremor is defined as an involuntary, rhythmic, oscillatory movement of a body part^1^. Action tremor occurs while voluntarily maintaining a position against gravity (postural tremor) or as the affected body part approaches a visual target (intention tremor)^1^. Various etiologies can underlie and in many cases—including the large group of essential tremors—the etiology remains obscure^2^. As a common pathophysiological substrate of action tremor syndromes, an altered oscillating activity within a cerebello-thalamo-motor cortical network was demonstrated by neuroimaging and electrophysiological approaches^3,4^.

Notably, the amplitude of postural and intention tremor decreases in the absence of visual information and on the contrary is amplified by an increase of visual feedback. This phenomenon was reported in different tremor conditions, encompassing physiologic tremor, essential tremor (ET), dystonic tremor and intention tremor in multiple sclerosis^5–8^.

Recently, modulation of tremor amplitude by adjustment of visual feedback was shown for target force tremor in a grip force task in patients with ET and dystonic tremor^8,9^. In this task the participants aim to approach a target bar by moving a second bar via a grip force sensor. Visual feedback manipulation involved altering the visual gain on the display, with low gain resulting in small spatial amplitude, and high gain leading to larger spatial amplitude. A “widespread visually-sensitive functional network” was found in fMRI to contribute to tremor severity in this context^9^. This target force tremor paradigm might therefore serve as a model for examining the pathophysiological basis of sensory feedback dependent modulation of action tremors.

Although the impact of visual feedback on action tremor amplitude is well known, it has not been examined yet whether other afferent feedback like auditory sensation has a modulatory effect on tremor amplitude as well. In this view, feedback about the movement in general would increase the tremor amplitude. This would raise the question of a common underlying mechanism modulating tremor amplitude dependent on any sensory feedback. Also, a potential role of multisensory integration for tremor amplitude modulation has not been examined yet. Simultaneously incoming sensory feedback could lead to an amplification of the tremor modulating effect compared to the unisensory condition since an alteration of neural activity has been shown in the context of multisensory processing^10,11^. To test this, we examined the modulation of tremor amplitude by manipulation of visual and auditory feedback exclusively and by the combination of both. Therefore, we partially replicated the paradigm by Archer et al.^9^ and added another feedback modality using auditory feedback.

Pupil dilation coincides with cognitive arousal due to activation of the sympathetic nervous system and the task evoked pupillary response is known to reflect the mental effort to perform a task^12^, which was also shown in persons with ET (p-ET) by our group^13^. Apart from mental effort, pupil diameter also increases during physical effort, thereby reflecting not only the actual intensity of the physical activity but also the individual perception of the effort^14^. In summary, pupil size mirrors the level of effort, which is invested in a task, irrespective of whether it is physical or mental.

The objectives of our study were (1) to determine if auditory feedback modulates target force tremor in p-ET in a comparable manner to visual feedback and to combined multisensory feedback and (2) to assess whether pupil diameter, as a marker for arousal and noradrenergic activation, is increased during high feedback conditions. We hypothesized that p-ET perceive a higher effort/arousal during the high feedback tasks, as reflected by an increased pupil diameter, independently of the type of feedback.

## Results

14 p-ET and 14 healthy controls (HC), not significantly different concerning age and gender, were included into the study (Table 1). While there was no significant group difference in age (U = 86.50, p = 0.147), the Becks–Depression–Inventory (BDI-II, U = 113.50, p = 0.003) and Schmahmann syndrome scale (U = 137.00, p = 0.013) revealed a statistically significant difference between p-ET and HC. The TETRAS score was significantly correlated with age (r = 0.566, p = 0.035), but not with the BDI-II score (r = − 0.145, p = 0.637). The Schmahmann syndrome scale total score was negatively correlated with age (r = 0.32, p ≤ 0.001). The TETRAS Score was significantly correlated with the Schmahmann syndrome scale score (r = 0.26, p = 0.005), this correlation however showed to be not significant (r = 0.08, p = 0.448) when including age as a partial factor in the analysis.

**Table 1:** Clinical characteristics.

Variable	p-ET		HC		
	Median	25th/75th percentile	Median	25th/75th percentile	p-value*
n	14		14		
Female [n]	6		7		n.s
Age [years]	63.00	[46.0/66.0]	65.50	[61.0/74.0]	n.s
BDI Score [n]	8.00	[5.0/11.0]	0.50	[0.0/1.8]	0.003
Schmahmann Modules [n]	2.00	[1/4]	1.00	[0.5/1.5]	0.013
Tetras Score [n]	41.00	[31.6/47.4]	–	–	–

*p-ET* persons with essential tremor, *HC* healthy controls, – not available, *n.s.* not significant, *Mann–Whitney-U test.

**Figure 1:**
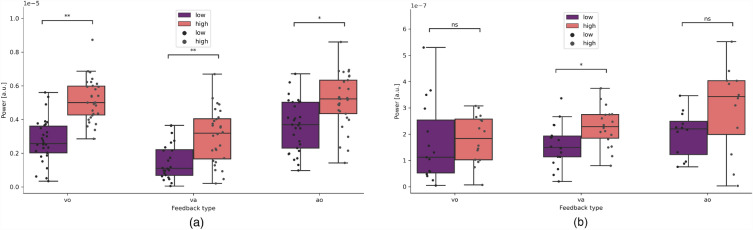
Single trial tremor force. (**a**) Trials of p-ET split per feedback type (visual only (vo), audio-visual (va), auditory (ao)) and viewing angle (low vs. high). (**b**) Trials of HC split per feedback type (vo, va, ao) and viewing angle (low vs. high).

The differences in force tremor between the conditions of high and low feedback were assessed. High and low feedback refers to the gain of visual and/or auditory feedback-signal, for more details please see “Experimental setup” in the methods section. The difference in force tremor between the conditions high and low feedback, as measured by the power spectral density (PSD) in the tremor relevant frequency spectrum (4–12 Hz), significantly differed between p-ET and HC in each of the feedback conditions (visual only (vo) (t[53] = 2.40, p = 0.018), audio-visual (va) (t[53] = 2.07, p = 0.041) and auditory only (ao) (t[53] = 2.71, p = 0.013, Fig. 1) (Supplementary Table 1).

p-ET showed a significant increase of force tremor during each high feedback condition (visual: p = 0.006; audio-visual: p = 0.005; auditory: p = 0.028). HC showed a smaller, but significant difference between low vs. high feedback only in the audio-visual condition (p = 0.048), but not in the other two conditions (visual: p = 0.09; auditory: p = 0.165).

**Figure 2:**
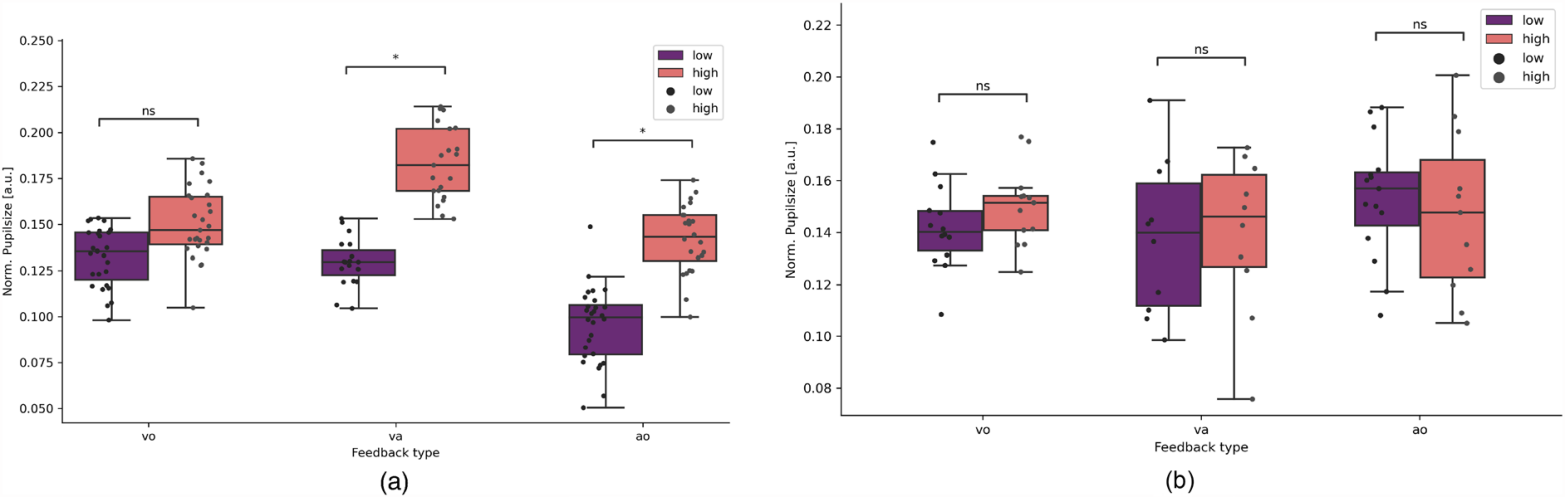
Single trial pupil size differences. (**a**) Trials of p-ET split per feedback type (visual only (vo), audio-visual (va), auditory (ao)) and viewing angle (low vs. high). (**b**) Trials of HC split per feedback type (vo, va, ao) and viewing angle (low vs. high).

Mean Force (MF), Unfiltered Force Error (RMSE, group: F(1, 294) = 2.857, p = 0.092 or feedback type: F(2, 297) = 1.671, p = 0.190.) and Force Power 0–3 Hz did not differ between conditions or groups.

p-ET showed a significant increase of pupil size during the high feedback compared to low feedback in two conditions (audio-visual: p = 0.039, auditory: p = 0.046), not however in the visual feedback condition (visual: p = 0.08, Fig. 2). HC showed no significant difference for pupil size between low vs. high feedback per condition (visual: p = 0.328; audio-visual: p = 0.167, auditory: p = 0.78).

Pupil dilation differences between p-ET and HC showed significant differences when comparing the feedback types for each feedback condition, visual only (t[53] = 2.00, p = 0.028), audio-visual (t[53] = 2.33, p = 0.022) and auditory only (t[53] = 1.33, p = 0.047).

## Discussion

In this study we investigated sensory feedback driven modulation of target force tremor amplitude in p-ET and HC.

In summary, we found that target force tremor amplitude is modulated by visual and auditory sensory feedback scaling in a comparable measure in p-ET. During the high visual, auditory or combined audio-visual feedback tasks the tremor amplitude was significantly increased. High feedback coincided with an increased pupil diameter in p-ET, but not in HC. Combined audio-visual feedback evoked the largest increase of tremor amplitude and pupil diameter in p-ET and additionally, a significant increase of tremor force in HC.

While it is well described, that visual feedback modulates action tremor amplitude in different underlying disease conditions like multiple sclerosis, ET and dystonic tremor^5–7, 15^, our study is the first to show that the amplitude of target force tremor in ET is modulated by a different quality of sensory feedback (i.e. auditory) in a comparable scale.

The increase of the tremor amplitude during the high feedback auditory-only condition cannot be explained by an increased error since the MF, RMSE and 0–3 Hz force power as markers for non-tremulous movements did not differ between the conditions or groups.

Our findings raise the question, whether there is a common underlying mechanism for sensory feedback induced tremor modulation in the context of different sensory qualities. A recent functional MRI study found -apart from the well-known cerebello-thalamo-motor cortical tremor circuit- a widespread visually sensitive network including key regions in the visual cortex and parietal lobule associated with alterations of essential tremor amplitude during visual feedback manipulation in a grip force task^9^. Interestingly, by the same group visual feedback-induced tremor exacerbation in patients with dystonic tremor was found as well, but in this patient group tremor amplitude modulation was not coupled to an altered BOLD signal of visual cortex regions^8^. Taken together with our finding that force tremor amplitude is comparably modulated by auditory feedback as well, this underlines the role of a common underlying mechanism for sensory feedback induced tremor modulation apart from the visual network.

Our finding that combined audio-visual high feedback evoked the largest increase of tremor amplitude in p-ET but also a significant increase of tremor in HC, underlines that the magnitude of sensory feedback per se correlates with a tremorgenic effect. A significant increase of finger tremor in healthy persons during postural pointing with augmented visual feedback was found in other studies as well^7^. This finding was explained by an increased motor unit recruitment and the resultant increase in 8–12 Hz EMG activity during augmented visual feedback. Our results suggest that the combined (va) high feedback condition had the most tremorgenic effect, in p-ET as well as in HC.

We hypothesized, that an increased arousal is associated with the intensification of the tremor amplitude. Recently, a modulatory role of cognitive effort during a serial seven task, as measured by a coincident pupillary dilation, onto the rest tremor network of Parkinson´s disease (PD) was shown^16^. This effect was most likely exerted by direct bottom-up noradrenergic influences onto the thalamus and indirectly by top-down cognitive influences onto the cerebello-thalamo-cortical circuit. Since the thalamus is a key node not only within the PD resting tremor network but also the action tremor network in ET as well^4^, an amplification of action tremor by ascending noradrenergic systems seems possible.

Enhanced feedback of any sensory quality during target driven physical tasks might increase the arousal/perceived effort level and thereby activate the ascending noradrenergic system, with the locus coeruleus (LC) as main effector^17^. Recent neuroimaging studies have confirmed a close relationship between the LC and bilateral thalamus and the cerebellum, both key regions within the action tremor network^18^. Therefore, cognitive arousal/perceived effort during motor tasks, induced by enhanced sensory feedback of any quality, might activate the LC-noradrenergic system and thereby mediate an amplification of action tremor amplitude via thalamic and cerebellar projections of the LC.

Therefore, in our experiment, pupil diameter was measured as a marker for cognitive arousal/perceived effort and an increase of pupil size during the auditory and audio-visual high feedback trials was found. Only during the modulation of the visual-only feedback there was no significant pupil dilation (although a non-significant trend). The most probable explanation for this finding is that visual stimuli elicit transient constrictions of the pupil (independently of an increment in light flux level on the retina) and thereby hamper the pupil dilation^19^. These "pupil motion responses “ are not present during the auditive only tasks because no moving object is displayed. Since external illumination and visual stimuli remained constant during the auditory feedback trials, pupil dilation occurred independently of external visual input. In the combined audio-visual tasks, the pupil motion response is most likely outweighed by the larger increase of arousal because of the combined feedback. The larger increase of arousal likely outweighs the pupil constriction induced by the visual target bar, thereby leading to a larger difference in pupil size between the two gain conditions. This is also supported by the finding that also in HC the largest tremor increase was found in the combined feedback condition.

In summary, it´s rather probable, that the pupil dilation reflects an increased arousal during the high feedback trials.

Another explanation for sensory feedback dependent tremor modulation could encompass the interaction between somatosensory cortex (S1) and the primary motor cortex (M1). M1 plays a crucial role as a feedback controller for motor control, performing dynamic updates of internal motor commands, which receive input from the somatosensory cortex (S1). However, as sensory feedback is changing for the same force output, such as in our paradigm where visual feedback is altered and does not match the applied force to the sensor, it might lead to conflicted processing of sensory information in M1^20,21^. This idea is supported by the fact, that S1 and the cerebellum are closely interconnected and work together during movement control^22^. Dysfunction of this interaction seems to contribute to the development of action tremor^23–26^. Therefore, understanding the complex interactions between M1, S1, and the cerebellum seems essential for understanding how action tremor emerges.

Our data of the pupillometry is intended as a primer of the LC activity^27^. Studies have shown that the LC projects into the thalamus and basal ganglia and acts as modulator of these regions. Both, the basal ganglia, and thalamus are involved within tremor generation^28,29^. In our task, two mechanisms might contribute to the fact that p-ET show a higher tremor force in high feedback conditions, modality independent. First, a bottom-up process triggered by the LC activity in a higher arousal state mutes down inhibition on subcortical tremor-generating structures. This is partially supported by our pupil data. Secondly, the cerebellum and sensoricortical structures integrate different sensory information (visual, auditory, and somatosensory) which are supposed to work as an efference copy for the feedback control of M1.

### Limitations

Our study has several limitations. The main limitation is that, by our experiment setup, we cannot finally prove that the altered effort/arousal (mirrored by pupil dilation) is directly caused by the enhanced feedback or is rather secondary to the increase of tremor. The enhanced arousal could also be just a secondary effect of the increased difficulty to perform the task with increased tremor because the participants need to correct more deviations. Although we cannot definitely rule this out, in this case we would have expected a correlation of the pupil dilation with the tremor severity (PSD in the tremor relevant frequency spectrum (4–12 Hz) or individual TETRAS scores) independently of the feedback condition, but this was not found.

Another limitation is, that the altered pupil diameter in the enhanced feedback condition might not reflect an altered arousal due to increased feedback or task difficulty but could be caused by the auditory feedback itself. Due to their tremor, p-ET heard more alterations in the tone. This might have caused an unpleasant sensation which might have induced the pupil dilation. Indeed, a pupil dilation in response to very pleasant or very unpleasant tones was shown in healthy persons^30^. However, when planning our experiment, we performed several pilot trials to ultimately find a pleasant sound volume and pitch for both, p-ET and controls. Therefore, it seems unlikely to us that pupil dilation in p-ET is caused by an unpleasant perception of sound, although we cannot fully rule that out. Another limitation is, that the feedback conditions were examined in a particular order und not randomized (see Material and Methods). Therefore, fatigue and attention may have polluted the results. The study intentionally omitted randomizing conditions to replicate a previous study accurately^9^. We always conducted the 'visual only' condition first to prevent fatigue or attention loss from skewing results. This approach might have influenced outcomes in subsequent conditions (audio-visual and audio-only). We accepted this limitation to compare the effects of the previous study with our initial visual condition. This decision balanced the need for replication accuracy with exploring auditory feedback's impact.

Depression is a prevalent finding in many p-ET^31^. Although our results show that most of our patients had no clinically manifest depression (median 8; scores from 0 to 9 reflect no depression, scores from 10 to 18 mild to moderate, and scores above 18 indicate severe depression), they had significantly higher scores than HC. In general, compared to non-depressed individuals, individuals with depression exhibit alterations of pupillary reactivity (either increased or reduced pupillary reactions) towards negatively valenced stimuli (e.g. emotive faces tasks)^32^. Therefore, depressive symptoms in our p-ET cohort might theoretically have interfered with our pupillometric findings.

Although p-ET and HC did not differ significantly in age, the percentiles show differences in age distribution of our cohorts. The effect of aging on task-related pupillary responses is still poorly understood and findings seem to be task dependent^33^. Therefore, we cannot completely rule out that age might have influenced our findings. Another limitation of the auditory feedback paradigm is that hitting the target tone might be easier (and therefore cause less arousal) for participants who are familiar with making music or singing. At least we excluded a manifest hypoacusis in all participants by a hearing test.

## Conclusion

In this study, it was found that the amplitude of force tremor in p-ET is modulated by alteration of sensory feedback, including visual and auditory, in a comparable manner. The auditory and audio-visual high feedback conditions were associated with a significantly larger pupil diameter in p-ET, possibly mirroring a higher level of effort/arousal invested in the task. Whether higher arousal/effort itself mediates an amplification of action tremor remains speculative. Further studies including imaging or high-resolution EEG might help to better understand the relation of feedback dependent tremor modulation in the future.

## Materials and methods

### Participants

The study was approved by the ethical committee of the Medical Faculty of Kiel (AZ 447/21) and was conducted in accordance with the Declaration of Helsinki. Participants gave written informed consent before participation. 14 persons with essential tremor (p-ET) and 14 healthy controls (HC) were included. All p-ET were diagnosed with ET by a movement disorder specialist, HC had no history of neurological or psychological disorders. All participants were right-handed and had no restrictions in vision or hearing.

p-ET were asked to pause tremor related medication and medication possibly affecting the pupillary motion (i.e. cholinesterase inhibitors, betablockers, benzodiazepines, caffeine) for at least 24 h. The clinical examination encompassed a complete neurological examination, a tremor assessment (The Essential Tremor Rating Assessment Scale, TETRAS^34^), a cognitive assessment (The Cerebellar cognitive affective/Schmahmann syndrome scale^35^) and the Beck´s depression inventory^36^.

Covariates between groups were compared using Mann–Whitney-U tests.

### Experimental setup

**Figure 3:**
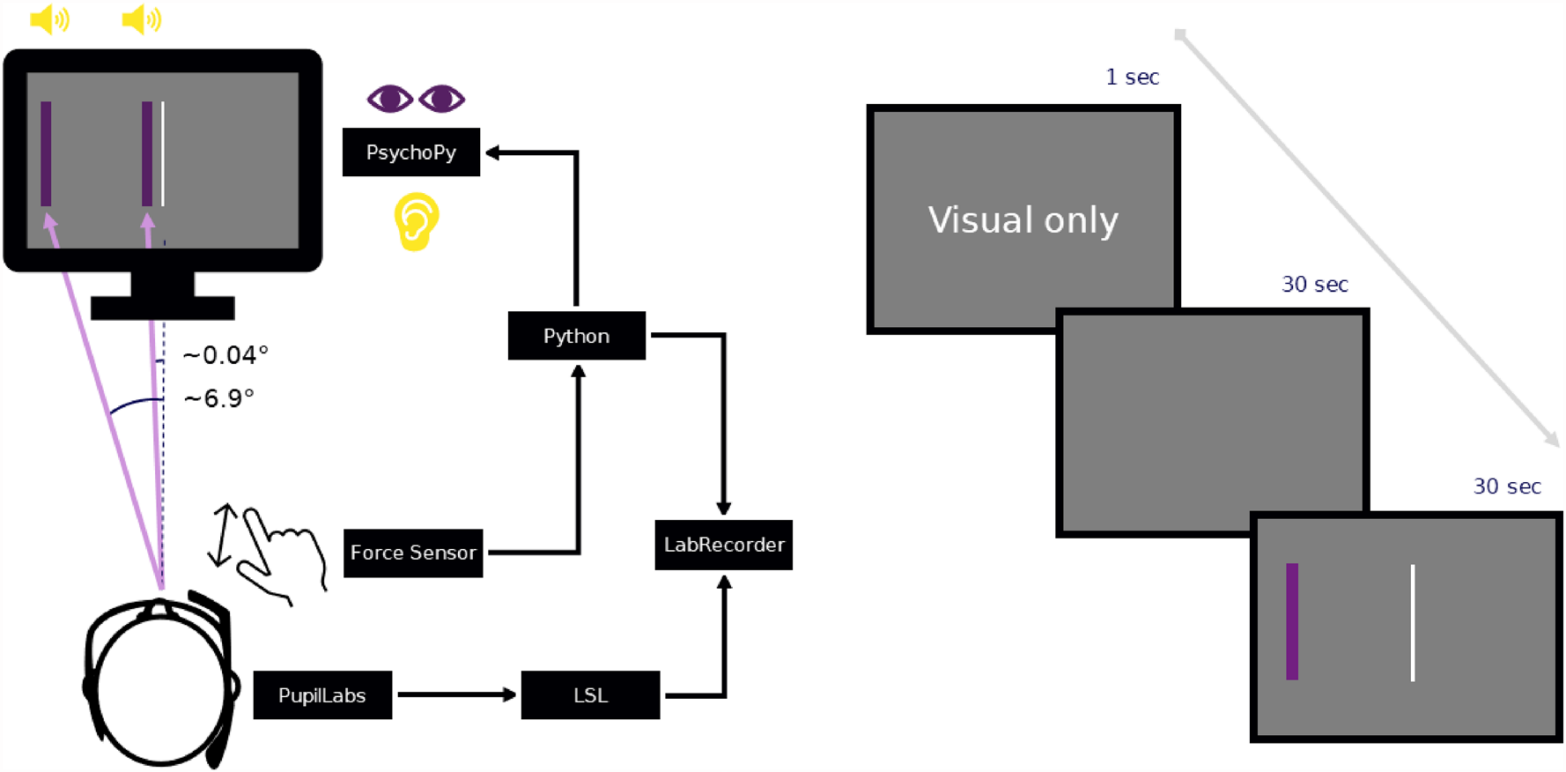
Experimental setup. (Left side) Datastreams (Force sensor, Pupil dialation and experimental triggers) are recorded via the Lab Streaming Layer. (Right side) Example epoch with timing of all elements.

In a computer-based task participants were asked to match a target force by using a force sensitive resistor (FSR). Feedback about the target position and the corresponding sensor was given either visually on a computer screen or auditory via headphones or with a combination of both. Force data were collected with a weight cell (Adafruit, ADA4541), which was connected to an amplifier (SparkFun, HX711) and digitized at 80 Hz via an ArduinoUno. The Arduino was connected via a serial port to the stimulus presentation computer. The experiment presentation was coded in PsychoPy (Peirce et al.,^37^) and presented on a monitor (LG24L600F, 144 Hz, 1920 × 1080 pixel). Inside the presenting script the data of the serial port (FSR) was used to feedback information of the applied force to the participant in real time (jitter delay < 10 ms) and simultaneously send to the Lab Streaming Layer (LSL) (Kothe et al.,^38^) for recording. Pupil data were recorded using a Pupil Core module (Pupil Labs, Berlin, Germany) with a sampling rate of 240 Hz. Calibration was done prior to the experimental task while data was send to LSL during the experiment via the Pupil LSL relay (Pupil Labs, 2021). All data streams (Experimental Marker, FSR and Pupil data) were recorded using the LabRecorder. For details see Fig. 3, left side.

### Experimental procedure

The experiment session lasted ~ 60 min and took place in a controlled laboratory environment. After participants gave consent to participate, clinical data and demographics were recorded. After this, they started the experimental task sitting in front of a computer (distance from the eyes to the screen: 90 cm). The experiment consisted of a training block and three subsequent experimental blocks, between which the subject could take short breaks. Prior to training the individual maximum force (MaxF) was determined. For this, participants were asked to apply maximum pressure to the force sensor with the thumb and index finger three times for 1 s. The maximum of the respective averages of samples was used as MaxF.

The task for the participants was to match a target force (15% of the individual MaxF) as quickly as possible and hold it for a period of 30 s. They got feedback on their performance during every trial in form of visual and/or auditory feedback. High and low feedback here refer to a previous study^9^ where different levels of feedback were introduced during a task to match a target force. Three different sensory feedback types were presented in the following order: 1. Visual only, 2. Audio-visual and 3. Auditory only. Visual only (vo) feedback consisted of a vertical bar which position varied depending on how close the target force was matched. The target bar and the force sensitive bar overlapped when the target force was matched. Auditory only (ao) feedback was provided by a reference tone (440 Hz) as a target and a second tone which varied in pitch depending on the distance to the target force (between 120 and 880 Hz). If the target force was matched, participants heard two 440 Hz tones. Audio-visual feedback was a combination of the vo and ao type. The performance between feedback types were assessed using the Root Mean Square Error (RMSE) and the amount of voluntary movement (Power 0–3 Hz). Each experimental trial consisted of a written cue what type of feedback is being presented, a 30 s resting period and a 30 s task period (see Fig. 3, right side).

In total every participant conducted 12 experimental trials, four of each feedback type. During each trial the feedback was altered using one of two factors applying different gain levels, resulting in different feedback conditions. The low gain (0.04°) and high gain (6.9°) resulting in a low or high feedback task to match the target force^9^.

$$Current \;position=\left({F}_{p}-{F}_{t}\right)*G+{F}_{t}.$$

Here F_P_ is the force produced by the subject, F_t_ is the target force, and G is the gain level used to manipulate the amplitude of feedback. The absence of force application kept the visual cursor static at the left side of the screen. As the participant exerted force, the cursor initiated a rightward movement. This movement was directly proportional to the applied force, culminating in the cursor's alignment with the target bar when the exerted force equalled the target force. Notably, the feedback mechanism incorporated variations which influenced the initial positioning of the cursor. Specifically, a reduction in the gain level resulted in a decreased travel distance for the cursor, thereby initiating its movement from a position incrementally closer to the target bar. Throughout the trial, participants were instructed to maintain the cursor's alignment as close as possible to the target bar.

Additionally, participants had to complete an auditory condition. This condition mirrored the fundamental principles of the visual feedback system. However, instead of a visual representation, participants were presented with auditory cues. In this setup, participants were exposed to two distinct tones. The first, a constant target tone, was set at a frequency of 440 Hz. The second tone varied in frequency, with its modulation being contingent upon the participant's force application relative to the target force. The frequency of this second tone was directly linearly related to the distance between the exerted force and the target force, providing an auditory representation of the participant's performance in aligning their applied force with the target force. In another condition, the participants underwent a combination of the visual and auditory feedback mechanisms at the same time. Participants were always presented the visual only (vo) condition first, followed by the combined condition (audio-visual, av) and the auditory only (ao) was always presented last. We applied a randomization within each condition for the different gain levels.

### Data processing

**Figure 4:**
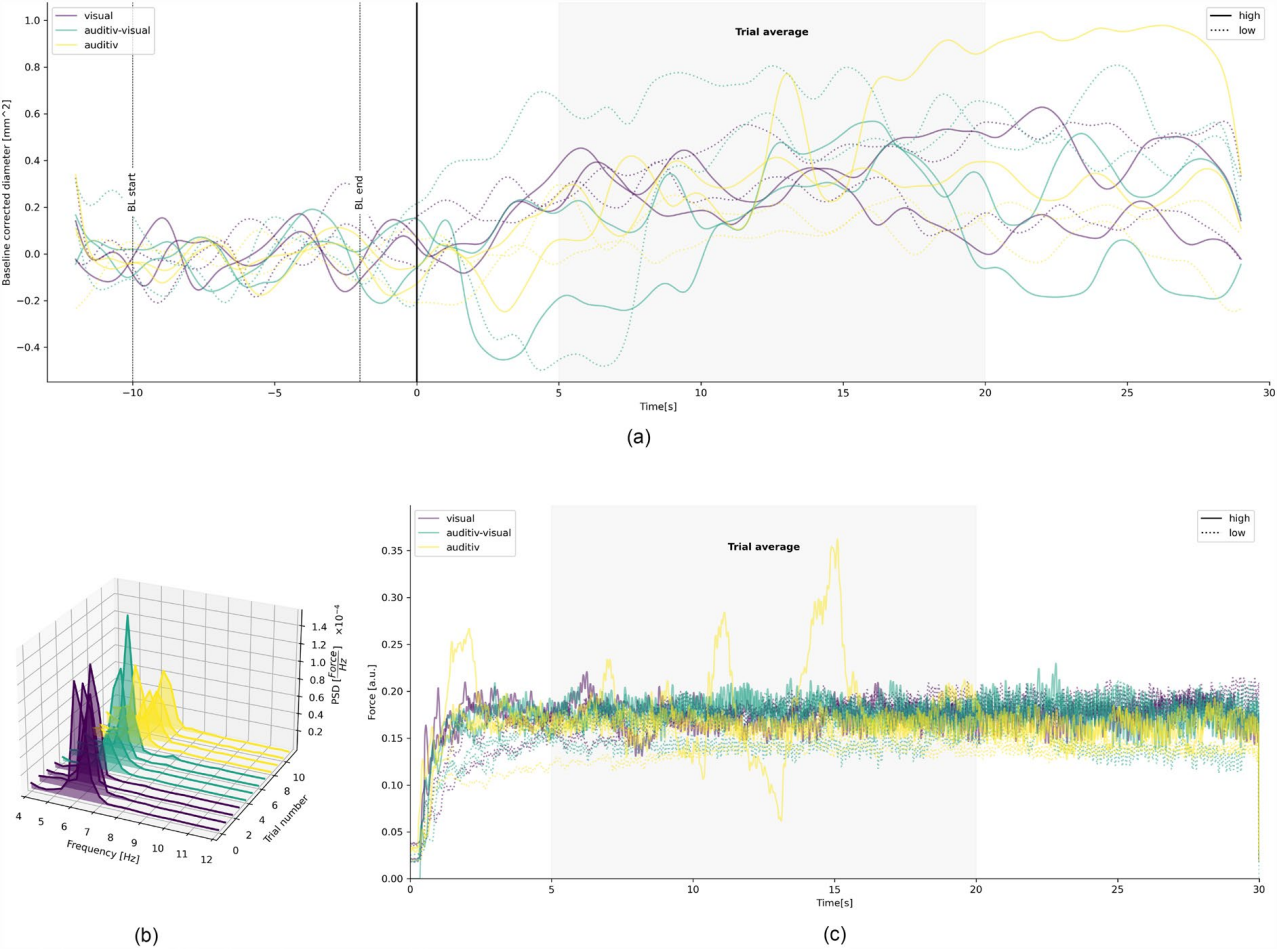
Single trial data of an exemplary participant. (**a**) The time course of the pupillometry is shown. (**b)** Single trial spectra of the force tremor per epoch. (**c**) Example of a participants single trial raw force data. Time courses are split per feedback type and feedback condition. One trial in the auditive only feedback conditions shows a stronger deviation to the target force, compared to other trials.

To ensure similar task conditions between participants, the force data was normalized to the participants MaxF prior during all trials. This was achieved by dividing every sample registered (applied force at each moment) by the MaxF * 0.15. Next, A fifth-order Butterworth band-pass filter was applied to the data with cutoff frequencies at 0.1 Hz and 12 Hz. The filter was implemented using the Second-Order Sections (SOS) format to ensure numerical stability and executed by the scipy package (1.8.1) in python (3.10). After filtering, data was cut into trials to estimate power-spectral densities, using the psd_array_welch function from the MNE package (1.3.1). For force tremor relevant power, a frequency window of 4–12 Hz was defined (the power spectral density (PSD) in the tremor relevant frequency spectrum (4–12 Hz)). For voluntary movement a 0–3 Hz frequency window was defined. Unfiltered force error (RMSE) during a trial was calculated by the root of the squared difference per sample to the target force. Figure 4 shows an example of time series data for pupil size, raw force data and spectra from the raw force data.

The pupil data was first cleaned of artifacts to be later averaged for the time window of interest. The initial step involved the identification and removal of blinks, which were detected based on anomalies in gaze acceleration and the deviations in the confidence score provided by PupilLabs software (version 2.5). Samples which were identified to be part of blinks were marked as 'Not a Number' (NaN) values within the pupil data time series. Following the identification of blinks, these NaN values were addressed through interpolation. This interpolation was conducted using a Fast Fourier Transform (FFT) convolution. The convolution employed a Gaussian kernel with a span of 120 samples, approximately equivalent to 0.5 s, utilizing tools from the astropy software package (version 5.1).

After cleaning the raw time series, data was cut into epochs. Each epoch underwent a subtractive baseline correction, from − 10 to − 2 s before the onset of the trial. This was possible as the 30 s prior to the trial start were a resting like condition. The baseline correction was applied to each epoch individually as suggested previously^39^. The mean of the artifact free and baseline corrected time window was used for statistical analysis. Preprocessing scripts of the FSR and pupil data have been made publicly available (https://github.com/JuliusWelzel/tremor_feedback_jw).

### Statistics

Clinical data were compared between groups using a Mann–Whitney-U test when not normally distributed based on Shapiro–Wilk tests. Correlation analyses were conducted using a Pearson correlation if Levene’s test and Shapiro–Wilk test allowed it, otherwise Spearman rank correlation was used. For the FSR data interindividual differences between means of the low and high feedback condition were calculated per feedback type. T-tests between groups for every feedback condition were calculated. The same was done for pupil size data. All statistical analyses were performed in Python (3.10) using the scipy package (1.8.1) or pingouin package (0.5.2).

### Sample size justification

The protocol was designed using a sequential design with maximal sample sizes to efficiently reach statistical power. With this approach, the study is conducted in stages with the aim of collecting the minimum number of participants required to achieve the desired level of statistical power from the study to replicate^9^.

The study was designed to have power of 0.80 from the original paper, therefore the data tested after each participant until the desired power level is reached. The desired power is achieved before the planned maximal sample size per group is reached (max n = 25), hence the study was stopped early, as higher numbers would have impacted the other outcome parameters to an unknown extent.

Overall, sequential designs with maximal sample sizes offer an approach to optimizing statistical power in replication studies where smaller changes to the protocol are made (Schönbrodt and Wagenmaker^40^).

### Supplementary Information

Supplementary Tables.

## Data Availability

The datasets generated during and/or analyzed during the current study are available from the corresponding author on reasonable request.
