# Supplementary material for: The interplay of sensory feedback, arousal, and action tremor amplitude in essential tremor
Source: Sci Rep. 2024 Feb 21;14:4301. doi: 10.1038/s41598-024-54528-5 (PMC10881477; doi:10.1038/s41598-024-54528-5)
Supplement: Supplementary file 1 — Supplementary Tables. [file 41598_2024_54528_MOESM1_ESM.docx]

**The Interplay of Sensory Feedback, Arousal, and Action Tremor Amplitude in Essential Tremor**

Julius Welzel^a^, Miriam Güthea, Julian Keilb, Gesine Hermanna, Robin Wolkea, Walter Maetzlera, and Jos S. Becktepea

aUniversity Hospital Schleswig-Holstein, Kiel, Germany

bDepartment of Psychology, University of Kiel, Kiel, Germany

*Correspondence should be addressed to J.S.B. (j.becktepe@neurologie.uni-kiel.de)

| Variable | p-ET | |  | HC | |  | |  | |
| --- | --- | --- | --- | --- | --- | --- | --- | --- | --- |
|  | low | | high | low | | high | |  | |
| Power [0-3 Hz] | 1.6±0.2e-06 | 4.6±1.7e-06 | | 3.3±5.5e-07 | 2.1±2.5e-07 | |  | |  |
| Power [4-12 Hz] | 2.6±1.2e-06 | 5.0±1.4e-06 | | 1.1±1.8e-07 | 1.8±1.0e-07 | |  | |  |
| RMSE | 1.7±1.2e-03 | 1.5±1.3e-03 | | 1.3±0.8e-03 | 0.9±0.5e-03 | |  | |  |
| MVC [%] | 14.9±1.7 | 15.0±1.4 | | 15.4±1.3 | 15.6±0.8 | |  | |  |
| **Supplementary Table 1a:** Values from experimental paradigm for the **visual only** feedback expressed as Mean±SD. p-ET = persons with essential tremor, HC = healthy controls. | | | | | | | | | |

| Variable | p-ET | |  | HC | |  | |  | |
| --- | --- | --- | --- | --- | --- | --- | --- | --- | --- |
|  | low | | high | low | | high | |  | |
| Power [0-3 Hz] | 2.6±1.2e-06 | 5.0±1.4e-06 | | 1.1±1.8e-07 | 1.4±1.0e-07 | |  | |  |
| Power [4-12 Hz] | 1.2±1.4e-06 | 2.9±1.9e-06 | | 1.4±0.8e-07 | 2.4±0.7e-07 | |  | |  |
| RMSE | 1.9±1.2e-03 | 1.9±1.6e-03 | | 1.3±0.5e-03 | 0.9±0.9e-03 | |  | |  |
| MVC [%] | 15.1±2.1 | 14.6±1.3 | | 15.6±1.1 | 15.6±0.8 | |  | |  |
| **Supplementary Table 1b:** Values from experimental paradigm for the **visual-auditory** combined feedback expressed as Mean±SD. p-ET = persons with essential tremor, HC = healthy controls. | | | | | | | | | |

| Variable | p-ET | |  | HC | |  | |  | |
| --- | --- | --- | --- | --- | --- | --- | --- | --- | --- |
|  | low | | high | low | | high | |  | |
| Power [0-3 Hz] | 2.6±1.2e-06 | 5.0±1.4e-06 | | 1.1±1.8e-07 | 1.4±1.0e-07 | |  | |  |
| Power [4-12 Hz] | 3.6±2.0e-06 | 5.2±1.8e-06 | | 2.1±1.2e-07 | 3.6±1.8e-07 | |  | |  |
| RMSE | 0.8±0.8e-03 | 0.8±1.0e-03 | | 0.8±0.9e-03 | 0.9±1.3e-03 | |  | |  |
| MVC [%] | 15.4±1.0 | 15.0±1.1 | | 15.3±1.3 | 15.1±0.9 | |  | |  |
| **Supplementary Table 1c:** Values from experimental paradigm for the **auditory only** feedback expressed as Mean±SD. p-ET = persons with essential tremor, HC = healthy controls. | | | | | | | | | |
